# Social Media Influencers in Equestrian Sport

**DOI:** 10.3389/fspor.2021.669026

**Published:** 2021-04-21

**Authors:** Aage Radmann, Susanna Hedenborg, Lovisa Broms

**Affiliations:** ^1^Department of Teacher Education and Outdoor Life Studies, Norwegian School of Sport Sciences, Oslo, Norway; ^2^Department of Sport Science, Malmö University, Malmö, Sweden

**Keywords:** equestrian sports, social media, knowledge exchange, authencity, intimacy

## Abstract

The study analyzes and explains the impact of social media influencers on stable cultures in Sweden and Norway, contributing to the understanding of the complex relationship between equestrian sports and social media—a relationship that is important for the welfare of horses. Since equestrianism is one of the most popular sports in Sweden and Norway, influencers' social media communication greatly impacts followers' perception of the human-animal relationship. Despite the popularity of equestrian sports, studies thereof are rare, and research combining equestrian sports and social media is almost non-existent, making this study important and relevant. The analysis focuses on the six biggest equestrian influencers in Sweden and Norway and their social media accounts. Goffman's (1967) micro-sociological perspectives, alongside previous research on social media, are used to discuss knowledge exchange, co-creation of authenticity and intimacy and sponsorship and advertisement. The influencers mainly focus their communication on horse-related issues, and their (and their followers) love of horses create the intimacy needed as a base for other messages (perceived knowledge and advertisement). The intimacy and authenticity are strengthened in the interaction between followers and influencers, and the latter receive positive feedback for their way of handling their horses' lives. The expressive order of stable culture(s) is seldom questioned and the acceptance of this order is likely to make the commercial endorsements less visible and more convincing.

## Introduction

Social media has changed communication dramatically; today, anyone can share anything at any time outside the gatekeeping function of traditional media and with the help of new, relatively cheap, technologies (cf. Bruce, 2016). In recent years, the influence of social media on the dissemination and quality of knowledge has become increasingly evident. With relevance for human well-being, this is exemplified by unscientific claims that the triple vaccination of children causes autism; claims that are widespread on the Internet and have had an adverse impact on vaccination rates (cf. Wang et al., 2019). Another example is misleading information about fluoridation (Wang et al., 2019). A third example is discussions about head lice in an online parenting community. Hine (2014) observed that scientific knowledge was often referenced, but not privileged above personal experience, and was sometimes seen as less credible than the latter. The development challenges traditional sources of knowledge and raises questions about how knowledge should be interpreted and valued.

This article aims to explore how social media influencers within equestrian sport perform and communicate with their followers in order to discover how ideas and perceived knowledge are formed and transferred in the sport. The study contributes to the understanding of the complex relationship between equestrian sports and social media—a relationship that is important for the welfare of horses. The concept of the influencer usually centers on what an influencer does. In this article we adhere to a more precise definition presented by Abidin (2015) who emphasizes that social media influencers accumulate followers on blogs and social media, presenting their everyday lives in text and pictures promoting products and services through advertorials (cf. Pöyry et al., 2019). Social media shapes our consuming behavior, not least through the actions of social media influencers. Stubb and Colliander (2019) note that influencers play an important role in consumption processes, as purchasing decisions are affected by the actions of others in our circle of acquaintances. Many people perceive themselves to have a close relationship with influencers, and are thus more likely be swayed by influencer marketing than by traditional advertising. Most often, influencer marketing is not perceived as advertising, but as recommendations from a friend (Stubb and Colliander, 2019).

We focus on equestrian influencers in Sweden and Norway. Equestrian sport is one of the most popular sports among Swedish and Norwegian girls and women. In Sweden, approximately half a million people ride regularly and 151,000 are members of the Swedish Equestrian federation in a population of 10 million. The federation encompasses ~900 associations, around half of which are riding schools. Riding is the third largest children's and youth sport, and the biggest para-sport (https://www.ridsport.se/Omoss/Statistik). In Norway, the Norwegian Equestrian Federation (NRYF) is the 13th largest sports federation with 30,000 active members in 340 registered clubs. Some clubs offer riding school activities, but most of the riding schools are not organized in NRYF. According to a survey conducted in 2014, clubs organized in NRYF only owned 16% (*n* = 220) of the riding schools, while the vast majority (73%) were privately owned, and 10% were owned by municipalities (Hatlevoll, 2014).

The interest in horse riding is not only evinced by the number of members in the two countries' equestrian federations, but also by social media activity. Numerous blogs as well as accounts on Instagram, Twitter, and Snapchat are devoted to horses and horsey people. Previous research shows that riders use social media for discussing, and arguing, about how best to take care of horses—not least since traditional media covers equestrian sports considerably less than major team sports such as football and ice-hockey (Dashper, 2017).

Despite the popularity of equestrian sports and the growing number of social media accounts connected to equestrian sports, research on the content of these accounts is scarce. However, a new study on perceptions of social media content demonstrates that young riders negotiate and contest their equestrian identities in relation to what their role models (i.e., influencers) post on social media (Broms et al., 2020). They express a problematic relationship to perfection—it is both admired and questioned in relation to what is seen as an authentic stable culture Hence, a study of equestrian social media influencers and their communication is important. Social media influencers are likely to impact followers' perception of the human-animal relationship as well as consuming behavior and can therefore affect the welfare of horses. In addition, a study of the social media influencers contributes to the larger field of social media and sport. Our research questions concern how influencers perform (i.e., present themselves and their everyday lives) on social media; what they communicate on social media; and how they, together with their followers, shape and determine what is and is not accepted in the stable culture(s) in cyberspace. Three research questions will be in focus in this study:

1. How do the equestrian influencers perform on social media?
2. What do the equestrian influencers communicate on social media?
3. How can the social media communication initiated by the equestrian influencers be analyzed and understood?

## Theoretical Frame

The present study uses a multifaceted theoretical framework inspired by Erving Goffman's (1967) concepts *face, face-work, line*, and *expressive order*, which Goffman identifies as main elements of a meeting. They constitute essential aspects of the interaction ritual and form the basis for social interaction. Face-work and line determine the atmosphere and the role that the interlocutors play in each given meeting. Face can be defined as the positive social value a person claims for themselves. To maintain the face, individuals participate in an interaction ritual process called face-work. Face-work can be explained as how an individual presents themselves to others in order to influence the interaction while evaluating the situation and performance of all participants over time. Line is the presentation of the self that others perceive, such as spoken language, gestures, and facial expressions (Goffman, 1967).

The fourth concept, expressive order, is related to what is allowed and the rules for the game plan in which communication takes place. In other words, people implicitly agree on what expressive order prevails (Goffman, 1967). People are constantly attentive of the expressive order and if someone breaks it a correction process occurs. That is, when a person has done something that results in a “wrong face” or “lost face” in a way that cannot be ignored, this must be corrected if meaningful communication is to be maintained. The rules of the game must be reset for the expressive order to apply again (Goffman, 1967).

Goffman's theories and concepts were developed long before social media existed but have nevertheless been of great importance for media research, and have proven fruitful for analyzing the new media landscape. Many contemporary media researchers, not least those who explore social media, are inspired by Goffman's ideas about social interaction, face-work, line, and expressive order and suggest that a study of online activities can “…offer opportunities to contribute to further developing the Goffman framework” (Bullingham and Vasconcelos, 2013, p. 110).

The study is inspired by media theory and how media research explain the development of media celebrities. As early as 1956, Horton and Wohl (1956) coined the term “parasocial relationships” to describe how TV and radio celebrities developed a one-sided interpersonal relationship with viewers and listeners to create an illusion of intimacy and friendship. Through small talk with listeners or viewers, direct accusations, close-ups, and sound control, a more intimate relationship was created with media consumers, who were thus more likely to continue following specific media productions. As a result, viewers perceived celebrities as real friends with whom they had close relationships. This relationship was not reciprocal; all communication and content were unidirectionally issued from the media houses to the listeners and viewers, who had no opportunity to respond personally or interact with the celebrities (Horton and Wohl, 1956; Forslid et al., 2017). The new digitalized media landscape has opened the possibility for two-way communication. While formerly celebrities remained separate and kept a distance from their fans, influencers build their popularity and self-branding on closeness and interactivity with their followers, gaining trust by responding to them. Thereby, followers feel a strong sense of closeness and an experience of friendship with the influencers (Senft, 2008; Abidin, 2018). According to Hirdman, people who are active on social media easily create emotional bonds of closeness and intimacy with social media celebrities, in other words, with people they do not know and have never met. These relationships can sometimes be experienced as stronger and deeper than those they have with people in their immediate environment (Hirdman, 2018).

## Previous Research

### Social Media

Definitions, interpretations, and understandings of social media have been discussed and changed over the past 20 years. Papacharissi (2015) claims that social media is a dynamic and context-specific term and that

…our understanding of social media is temporally, spatially, and technologically sensitive—informed but not restricted by the definitions, practices and materialities of a single time period or locale. How we have defined social media in societies has changed and will continue to change (Papacharissi, 2015. p. 1).

Researchers have used overlapping concepts for social media, SNS (Social Network Sites), and OSN (Online Social Networks). The concept SNS was dominant in the period 2003–2008 and OSN between 2009 and 2014 whereas social media has been the most frequently used overall term (McCay-Peet and Quan-Haase, 2017). Sloan and Quan-Haase indicate three characteristics of social media based on a synthesis of the participating researchers' definitions thereof. Social media…

“…Have the capability to support user-generated content in forms such as images, text, videos, and statuses…”
“…Provide a means for users to connect with one another…”
“…Support various means for members to engage with one another in the form of collaboration, community building, participation, sharing, linking, and other means…” (Sloan and Quan-Haase, 2017, p. 5).

In this study, social media will be used as an overarching concept encompassing the various media platforms studied, all of which fit the definition suggested by Sloan and Quan-Haase (2017).

Sport and social media have developed in symbiosis as a result of technological developments. The new media landscape creates new sports such as e-sports and exergames, and new ways of communicating and interacting through and about sport such as self-tracking and digital fan communities. Previous research has demonstrated the importance of studying the political and economic impacts of social media on sport, including the new roles of media producers and the diversification of production, changes in consumption as an effect of social media, and the effect of social media on gender roles in sport (Billings and Hardin, 2016; Ross and Rivers, 2020). Media research has given considerably greater attention to some sports and events than others: for instance, there is a substantial body of research on football and the Olympic Games. However, the effects of the new media landscape must be studied in relation to a variety of sports, as different sports may exhibit differing patterns of online participation. A study of media representations of equestrian sports in the mid-20th century showed that female riders were presented as real athletes far earlier than women competing in many other sports (Hedenborg, 2009). Hence, a study of different sports and their relation to social media contributes to new ways of thinking, interpreting, and understanding the development.

So far, research on social media and equestrian sports is scarce. Dashper (2017) has analyzed the content of blogs by English hobby and elite riders. Inspired by auto-ethnography, she reflects on how she herself disregarded her veterinarian's advice and Googled to find information when her horse fell ill. Dashper's study shows that Internet forums are important as spaces for narratives about horse-human relationships and for creating standards for horse-keeping. Studying Finnish blogs, Schuurman (2014) shows how relationships between horses and humans are created in stories where community, meaning, and joy are combined for both parties. These stories are not necessarily based on scientific knowledge about how horses act and react. Instead, they are built on an idea of horses and humans having emotions in common. Byström (2015) analyze how safety is addressed on 32 Swedish social media platforms (mostly blogs) for leisure riders. Their study shows that safety is rarely emphasized, and when it is, it is primarily the safety of the horse. Furthermore, Byström (2015) demonstrate that safety had different meanings for riders depending on riding style, experience, and ideal image. Since advice posted online can be more or less reliable, the authors stress that inexperienced riders should not blindly rely on it.

### The Influencer

In her studies of influencers Abidin (2018) indicates that traditional media celebrities practice a sense of separation and distance from their audiences. Microcelebrities, like influencers, differ from the traditional ones as their popularity is premised on feelings of connection and interactive responsiveness with their audience. They are expected to be “real people” with “real issues.” In addition, microcelebrities are more strongly obligated toward their audiences than traditional celebrities, as their fame is co-constructed through a community of interested viewers (Abidin, 2018). The influencer economy has a large turnover and is an increasing monetary power in the media landscape. It is difficult to compare the turnover of the influencer economy in different countries as such figures can be presented in different ways. The Swedish Institute for Advertising and Media Statistics (IRM) reports that the Internet accounted for 10.9% of total advertising investments of 81 billion SKR in 2019 (Institutet för reklam mediestatistik, 2019) According to IRM, the total advertising investments in Norway amounted to 29.3 billion NKR in 2019, the Internet accounting for 10.5% (Institutet för reklam mediestatistik, 2019). This industry has innovated the labor market as well as how people work (Abidin, 2018). Abidin claims that influencers generate income in three ways: from advertorials, which are highly personalized; selling advertising space on their blogs and social media platforms (each influencer presents their own rates and package deals); and selling wares, with some influencers starting their own brands (Abidin, 2018). In addition, Abidin discusses the shift in the ecology of influencers from perfect and tasteful images to an amateur-aesthetic and performative authenticity, a “calibrated amateurism” (Abidin, 2018). In a study of influencer marketing strategies, Audrezet et al. (2020) elaborate influencer authenticity further and states that there are two different forms of authenticity strategies: passionate and transparent authenticity. In the first one, the influencers are driven and directed by their passions, interests, and what they believe in, rather than earning money. These influencers are intrinsically motivated. The passionate authenticity stands in contrast to transparent authenticity, which is extrinsically motivated and takes its starting point in a product or company idea, and highlights the content of the products. The strategies are described by Audrezet et al. (2020) as four paths: absolute authenticity (passionate and transparent), fairytale (only passionate), disembodied authenticity (only transparent), and fake authenticity (neither passionate nor transparent).

Previous research on sport and influencers is scant, yet a growing field, not the least in relation to attempts of measuring impact through for example number of followers. This has, however, been problematized. In a study of sports influencers on twitter Lamirán-Palomares et al. (2020) used three dimensions to assess influence: activity (number of tweets and outdegree), authority (retweets and PageRank) and popularity (number of followers and indegree), and demonstrated that authority was the most influential dimension and the accounts with the greatest influence on Twitter turned out to be those related to organisation of the event and those of the athletes taking part (Lamirán-Palomares et al., 2020).

In what way influencers are accepted or not within the physical culture has been studied too. In a study of social media influencer labor and the construction of identity in bodybuilding Wellman identifies tensions between body-builders, trainers and influencers. The latter see themselves as productive workers and as important parts of the bodybuilding culture, whereas trainers and bodybuilders discredit their digital labor (Wellman, 2020).

## Methodology

Influencers' posts and comments on social media constitute the main source material for this article. As a complement, published interviews and books by influencers are used. We began by identifying Swedish and Norwegian Instagram accounts of influencers presenting themselves as connected to the equine sector, with taglines such as “international showjumper” and “Bridleless dressage, liberty, tricks.” A study of Instagram accounts was motivated by the fact that this social media platform is of great importance for today's influencers (Lee and Eastin, 2020). We subsequently selected the influencers with the highest number of followers from each country, as a high number of followers indicates that these influencers reach many people, which suggests they have a greater impact than others. Three influencers from each country were selected as there turned out to be a significant gap in the number of followers between the three most popular accounts and the subsequent ones. The influencers we have studied were between 18 and 27 years of age, and include five women and one man. The number of posts varied between the influencers and Table 1 shows that Vilde and Katrine posted the least during the studied period. Since they also have less followers than the others, we decided to concentrate our analysis on the four influencers with the highest number of followers.

**Table 1:** The six most followed influencers on Instagram in the equine sector in Sweden and Norway.

**Pseudonyms**	**Nationality**	**Female/Male**	**Age**	**Number of followers**	**Number of posts on Instagram during period 1 and 2**	**Social media**
Bella	Swedish	Female	18	364,000	49 and 47	Instagram, Blog, YouTube
Elin	Norwegian	Female	21	237,000	21 and 17	Instagram, YouTube
Fredrik	Swedish	Male	27	158,000	23 and19	Instagram, YouTube
Rebecca	Swedish	Female	24	92,800	83 and 63	Instagram, Blog, YouTube, FB
Vilde^*^	Norwegian	Female	–	36,000	8 and 4	Instagram, YouTube
Katrine	Norwegian	Female	18	8,000	7 and13	Instagram, YouTube

*Followed during the period 9/10–9/11 2018 and 1/6–30/6 2019. Presented according to pseudonyms, gender, age, number of followers, number of posts and social media*.

* *Vilde does not present her age, but seems to be below 20*.

The number of followers ranged between 8,000 and 364,000. The equestrian influencers have a high number of online followers in comparison to traditional press. The largest newspaper in Sweden, Dagens Nyheter, has 129,000 Instagram followers^1^ and the leading tabloid, Aftonbladet, has 172,000 followers^2^ on its Instagram account. However, there are Swedish sports influencers with significantly higher numbers of followers, such as the football player Zlatan Ibrahimović with 45.7 million followers.^3^

This study abides by Norwegian and Swedish legal and ethical requirements and our data satisfies the policy of research ethics committees (Beninger, 2017). Yet, there are several ethical considerations in online research. In relation to personal data, influencers (and sometimes their followers) are easy to identify. We have chosen to pseudonymize the influencers (see Table 1), but are aware that additional data (books, articles, et cetera) makes it possible for readers to identify their real names. As we see it, the influencers have chosen to be public and are aware of (and work with) their public personas. However, all of the influencers are not equally public, which motivates our use of pseudonyms. Referring to individual followers is ethically more problematic, and therefore we have chosen to omit followers' screen names completely. When the influencers advertise products, we have chosen to replace the names and websites of the companies with “xxxx” so as not to increase the commercial exposure of the brands in question.

The influencers in this study use social media for broadcasting as well as networking purposes (cf. Larsson and Olsson, 2016). Apart from Instagram, the studied influencers used YouTube, Facebook, and blogs. Bella, the most popular influencer in our selection, used several interlinked platforms to communicate with her followers. The other platforms were also studied.

This study is performed using a netnographic method (Berg, 2015). The influencers' posts (writings, pictures, films) and comments on these posts were read and documented in a field diary every day for 2 months (2018-10-09–2018-11-09 and 2019-06-01–2019-06-30) using thick description (Geertz, 1973). As equestrian sport is partly an outdoor sport, we chose to study social media posts from 2 different months (one in autumn and one in late spring) so as to analyze whether seasonal activities affected the influencers' performance. However, except for the influencers wearing more or less clothes, the weather, and the horses' equipment, we found no differences in the style or content of the influencers' communication. Our documentation included motifs of the pictures or videos, number of likes on a specific post, and the influencer's posted captions. In addition, we documented followers' comments (numbers and content). A typical observation of an Instagram post could be:

Picture: The picture shows a gray horse outside a stable wearing a saddle and bridle, but no rider is seen (6,959 likes for this picture).
Caption: Swedish championships
13 comments including “always inspiring”; many good lucks; good looking breeches; where is your helmet from; cheers; are you still sponsored by XX.

After documenting the posts, the field diary was entered in the computer-assisted qualitative data analysis software NVivo. We read and re-read the material, and performed an abductive thematic analysis. The above observation rendered notes such as: equestrian sports on a national level; competition logic; comments suggesting that the followers like the influencer/rider; and comments related to consumption. In other posts, more personal details were shared, e.g., this Instagram post:

Pictures: The influencer with her boyfriend in several different poses (6,721 likes for these pictures)
Caption: Is it OK to be jealous? Where is the line? //.// You have been able to comment on this and now I have the answer (the influencer refers the followers to her BlogSpot).
8 comments including hearts, “love you,” “goals couple”

The pictures show the influencer and her boyfriend: young, white, smiling, and wearing fancy clothes. This post was coded as personal, but we also noted that the influencer wanted her followers to continue reading on another platform (we also read the posts on the other platforms).

After our initial notes, we began coding the material, endeavoring to remain open to the observations. For the two examples presented above, we used codes such as “professional within a competitive sphere,” “asking for advice on what to buy” (for the first post) and “advice on private life” (for the second post). Three main themes were subsequently identified: creating authenticity and intimacy, horse life, and advertisement and sponsorship.

At this stage in the analysis, the theoretical concepts were introduced. In the first example, the expressive order of a specific context is outlined. In relation to the concept line, we interpreted the post as including a competitive equestrian sports context which the followers recognize and support. In addition, the influencer is asked where one buys certain equipment (helmet and, possibly, breeches); this is connected to commercialization. In the second example, the couple's line indicates that they are heterosexual, happy together, and rich, and as the influencer refers her followers to a previous discussion about jealousy and tells them that she has the answers, she presents a face in which to be trusted in matters related to personal life choices.

## Findings

### Creating Authenticity and Intimacy

In the following section, face-work, line, and expressive order will be discussed in relation to the influencers' online performance. Bella, the influencer with the highest number of followers in our study, explicitly communicates on Instagram that she sees herself as a professional blogger and influencer. She also describes herself as a “professional showjumper.” The second most influential influencer in this study, Elin, presents herself differently. She highlights the names of her horses and that she prefers dressage, tricks, and liberty dressage. Fredrik, the third most followed influencer in our selection, describes himself as an “athlete,” indicating that he views his involvement in the equestrian sphere as professional. Furthermore, Fredrik underlines that he loves his dog. The love of animals is also found in the fourth most followed influencer's presentation. She writes that she loves horses, but also connects herself to the professional showjumping context in describing herself as “jumping from baby heights to 155 cm.”

Bella, Elin, and Fredrik use several different social media channels and they also present themselves in interviews in traditional media, books, and on their own social media sites. Bella connects herself to the influencer sphere more expressively than the others. She underlines that she is a professional influencer in her Instagram posts:

“I have to give myself time to review emails and to blog. It IS my job, and it must be taken care of. It's one of the most fun things I know” (20190607).

Bella has a long history of being an influencer. She started blogging at the age of 9, reaching a wider audience by participating in an equestrian TV show when she was 12 years old. She has also had her own reality TV show. In addition, she has published two books about herself geared toward children between the ages of 12 and 15 (Berntsson, 2014, 2016). In these books, Bella describes her life, telling the reader that she started out as a shy, friendless girl with a stutter. She thanks her family for their support, and tells the reader that it took time for her to begin to like horse riding and that she began riding at a riding school, receiving her first pony at the age of 8. The reader is also introduced to Bella's horses. She writes that the horses have become her closest friends together with her followers on social media. The presentation of Bella's relationship with her horses is important for her followers' perception of her and the formation of trust between her and them. Her face-work creates a specific kind of authenticity and intimacy as she and her followers presumably share interests.

Bella writes that it was her idea to start a blog, and that her parents were hesitant, initially demanding to survey her posts. Her blog grew quickly and was published on the site of one of Sweden's biggest free journals.

“Now I have on average 200,000 readers every week and have the biggest horse blog in Sweden and the biggest blog for teenagers in the Nordic countries. I have 400,000 followers on Instagram, 30,000 followers on Snapchat every day and a YouTube channel with 80,000 followers and 17,5 million viewings of my films.” (Berntsson, 2016, p. 14)

Bella tells the reader that she receives countless questions every day that she tries to answer and wants to be a friend to anybody who follows her. The blog is described as “…my way to try and help others…” (Berntsson, 2016, p. 26). At the same time, she tells the reader that she has been heavily criticized for her style of writing, being told that she is too sloppy. The book, which is co-written, is stylistically more formal. Bella problematizes this fact, stating that on social media she wishes to be quick, and wants her readers to feel that she writes to them in the manner of a text-message. She also gives advice in her book, centering on the idea of standing up for oneself, like Bella feels she has done. She describes herself as having had to fight to get to where she is today, and underlines that anyone can succeed—if they work hard enough.

Bella often addresses private and personal questions with her followers, whose responses are immediate. While she presents herself as a horse person, her posts on horses are interspersed with personal comments and pictures from her more private life, such as what she had for breakfast or what she did last night with her boyfriend. A typical observation in the field diary of a Bella post is:

Picture: Bella and her boyfriend are dressed in fancy clothes. They are standing on asphalt, in front of a hedge and many pink flowers. Bella's boyfriend is standing straight in front of the camera, Bella is standing in front of him. He is wearing a dark suit, fine shoes, a champagne-colored tie, a button in the jacket is buttoned. His hair is fixed to the side. He has one hand in his pocket and the other on Bella's lower back. He laughs into the camera. Bella stands with her left leg in front, has her hand on her hip with her elbow out and straight back. She has black shoes with high heels and a champagne-colored-wrapped dress with large sleeves that end a little above the knees. She is wearing earrings; a necklace and her hair is in a tuft. She has her face turned toward the camera and smiles a little crookedly (20190606).

Bella is in constant communication with her followers. She asks for her followers' opinions and discusses their views with them. Bella sometimes posts controversial statements about private life, such as when to wake up in the mornings, how often anyone should wash their hair, or monogamy. These issues provoke her followers at times and can be seen as examples of the followers' resistance. An example is when Bella posts on her blog that she finds it offensive when people wake up as late as 10 A.M. on a weekday (20191012). Some of the followers say that they agree, others respond angrily that she does not know enough about their lives to generalize in this way. Bella answers that she has the right to an opinion and underlines that she thinks that people are lazy if they sleep during the day. “Even if they don't have a job they ought to get up and do something,” she says. She subsequently receives even more comments, pointing out that some people need to sleep during the day as they work late at night, and that according to others “night owls” are more intelligent. Another follower references a person who was bullied at work and did not feel well. Bella handles this critique by saying that she thinks it is “soooo boring to post her authentic opinions” and advices her followers to read more of the blog post than the heading. Some of her followers agree with her and show pity, but one responds that Bella will lose one of her followers. At this time in the communication, Bella has stopped answering and posts on an equestrian sport issue instead. The communication about when to get up in the morning can be interpreted as an example of Bella breaking the expressive order, to speak with Goffman (1967). It is, however, questionable whether the followers' resistance can be seen as part of a correction process and therefore another interpretation is that the expressive order is reinforced rather than broken. Bella refuses to get influenced. She continues to argue in the same way, even when her followers criticize her. A certain concession—or a reinforcement of her status as an influencer—may be traced when she encourages them to read the full post, not just the heading in which she expresses her disgust. The conflict is resolved in another way than restoring face. Some followers threaten to stop following Bella, and she starts posting about something else.

In other issues related to Bella's private life, she receives complete support and constructs intimacy with her followers through conversations around personal topics. An example of this is when she asks her followers:

“Have you been through a period of life that has changed you or your way of feeling?” (20190608)

Her followers are engaged and share their experiences, including difficult ones. They encourage and support Bella, as epitomized by a comment from a follower: “you are so tough—that is so honest.” In addition, Bella's followers comfort her by confirming that she has had difficult experiences that have made her tough and that she is inspiring and brave. The followers give different advice for what Bella can do to change the situation. One follower writes: “should you not hire someone to help you out, and not work yourself to death, even if it's fun?”.

Bella seeks comfort too:

“I do not want to whine or complain. But it's tough sometimes. You need a hug, a pat on the shoulder where someone randomly says, ‘you are best xx'. There you see how a few words can change your day.” (20190609).

Again, Bella receives supportive feedback saying she is honest and a good friend. She does not only seek comfort; she also gives advice on what is important and how her followers should handle life:

“I often get the idea of what I myself am impressed by in the sport. What makes me get goosebumps. Well, it's a development, when I see a development in a crew /… / When a crew has done that insecure, step-heavy work and stands there at the top of the podium. They really make me kneel in admiration.
-I like the journey, the crooked journey.
-It is hard work, and I am proud of that. Bloggers, influencers or whatever you are called. I am good at what I do, and there are hours of drive, determination and passion behind it.
-Find what you are passionate about, girls and boys. Put your heart there and you will create magic” (20190701).

In these posts Bella, tries to engage her followers to make their dreams come true, using herself as a role model and presenting herself as a prosperous influencer who is successful both in her private life and within the equine sector. Through the creation of intimacy, Bella presents herself as a “real person” with “real issues,” in congruence with what Abidin (2018) has indicated as distinguishing influencers from traditional celebrities.

Bella is not alone in presenting herself thus. The influencer Elin presents herself as an equestrian blogger, and claims to reach over 200,000 horse lovers world-wide every day. Just like Bella, she constructs herself as a real horsey person. She underlines that horses have been a part of her life since she was 7 years old. When talking about her life, her narrative appears to parallel with stories presented in children's horse books. She says that she first saw her dream horse at the age of 15:

“…met a young stunning Friesian gelding, seeking my attention in a field …”
(Matilde, 2020)

A few weeks later “…the curious black beauty became my own.” She continues the story, saying that she and her horse grew close and that they have developed an “unbreakable bond.” As in many stories in horse books for children, the story of Elin and her horse is not only a happy one (cf. Hedenborg, 2006). She tells her readers that she and her horse were “two insecure souls not sure about what to do about anything.” The narrative is happily resolved with Elin and the horse learning from each other and practicing riding “based on equal trust and light signals.” She presents the frames of another expressive order than the one presented by Bella. She has another face and line, but the relationship with horses is central. Elin also communicates that she is grateful to be able to help others to reach their goals with their horses and that her

“…goal is to inspire other bloggers to dare walk in another direction, in order to fulfill their own dreams”

Fredrik has a somewhat different approach than Bella and Elin, although a horsey authenticity is still central in his self-presentation. While they present themselves as serious, he jokes about himself on social media and in interviews, reflecting on horse life. In an interview from May 2019, he comments on his own posts on Instagram;

“Yes, @ (Fredrik) always has fun, is always happy with a twinkle in the eye. In @(Fredrik) world, it is almost always gold and green forests, says (Fredrik) about his Instagram persona” (Kentaur magazine, 2019).

There is a hint of irony in Fredrik's remark, suggesting he feels that the perfection displayed in his posts is not reflective of his real life. In the same interview, he claims that “the real Fredrik” is insecure and doubts himself. Like everyone else, he stresses, he has bad days—he is disappointed when his young horses do not qualify for competitions, or when he does not achieve the desired training results. Bullingham and Vasconcelos highlight that social media participants, although they generally reproduce their offline selves online, will not show their offline identity in its entirety. Instead, they choose specific aspects of their personalities (Bullingham and Vasconcelos, 2013). It is likely that none of the influencers we have studied show their whole offline identities. An interesting aspect of Fredrik's online self-presentation is that he constructs authenticity by telling his followers about his failures. In an interview in June 2019, Fredrik claimed that he has many followers because he allows them to see his whole identity, including both failures and successes:

A combination of many things! I share my journey; I think many people find it fun to get to know me and my horses and follow both success and adversity. I think it is important to share the whole picture, so it will be interesting and credible (Min Häst, 2019).

Bella, Fredrik, and Elin perform face-work in which a horsey authenticity is central. Through dialogues and reflections, they present themselves in a way that allows their followers to feel there is intimacy and closeness in their connection. In their posts, these influencers construct a sense of community and a common understanding of how life as a (horse)person can and should be lived. The line consists of successful people. Even when presenting failures, the influencers show themselves overcoming these, demonstrating to their followers that they are able to handle difficult situations. By showing followers (seemingly) authentic everyday life events, the influencer-follower bond is strengthened. Within the expressive order of the stable culture, which includes a horsey authenticity, intimacy, and perfection, there seem to be few disagreements. The combination of authenticity and perfection may seem contradictory, yet, the followers mostly assent to what they see online and do not dispute the influencers in relation to their lives with horses. However, Bella sometimes starts discussions, leading to debates around the expressive order and how she and her followers live their private lives. In these conversations there is sometimes resistance from the followers. The correction processes, as described by Goffman, are more difficult to trace. Bella's solution to the conflict seems to be to move on to another issue and for the followers to (threaten to) leave the influencer. Rebecca does not present herself as an influencer, nor does she reflect on herself as such. Her facework is different and more connected to hard work with her horses, which will be discussed in the next section.

Although our analysis of how the influencers relate to advertisement and sponsorship will be discussed further below, we wish to introduce Audrezet et al.'s (2020) concepts related to authenticity and marketing at this juncture, We see Fredrik, Rebecca, and Elin as examples of absolute authenticity. They oscillate between being passionate and transparent. Bella's case is more complex. Some posts can be interpreted as representing a passionate authenticity, while others clearly contain transparent authenticity, but there are also examples of disembodied authenticity.

### Horse Life

The emotional bonds between the influencers of this study and their followers are strengthened through a shared love for horses. In this section, focusing on equestrian sports, we will analyze the influencers' horse lives and knowledge exchange on horses and the welfare of horses. Rebecca, Fredrik, and Bella belong to an equestrian sports culture in which horses are bought and sold, trained for competition, and cared for when ailing or injured so as to return to performance. In their posted pictures, the influencers are often seen riding the horses wearing traditional riding gear: breeches, boots, helmets, and gloves. The horses wear blankets, saddles and bridles, and different kinds of leg protectors. Care of horses is also shown. Horses are brushed, led to the paddock, and receive different kinds of treatment from experts in the equine sector.

As described above, Bella presents herself as someone with long experience of various types of horses, and who can answer different kinds of questions about horses. She expresses her equestrian identity through content related to horse experiences—for instance, what she did in the stable this morning, descriptions of horses and how they are cared for, how a training session went, and what happened during a competition.

Picture: Bella dressed in competition gear in a show-jumping ring.
19,128 likes
Caption: Fucking hell I'm sharp as a rider here. After being hit many times, and with a heavy backpack and success drawn from under my feet I'm going to be as good again….
43 comments relating to questions on how to build self-confidence.
(20181106 Instagram)

Aside from asking her for advice on how to build confidence, Bella's followers support her. They tell her that she is great and that she inspires them, asking her for exercises that they can perform with their horses. Bella gives advice on how to take care of horses in her books as well as on social media. In addition, she discusses the difficulties of being a professional equestrian in relation to buying and selling horses:

Picture: A horse and rider jumping over an obstacle in a competition. Audience in the background.
7721 likes
Caption: Everything in life was balanced with you included! Miss u every day.
43 comments, including questions on why Bella sold the horse.
(20181015 Instagram)

A discussion of why horses are sold or not arose between Bella and her followers in the comments. One follower asks: “Why did you sell XX (horse's name) if you miss her so much?” Bella answers: “Please post a respectful question; as a wise person once told me, to miss someone is a nice sign that somebody is close to you—everything has its time.” Another follower asks: “Can't you buy her back? I would if I missed my horse so much!”

Bella answers: “That is not how it works. These kinds of questions tire me out.” This discussion shows a potential conflict between what is seen as “real” vs. inauthentic love of horses. Bella's authority and love of horses is questioned by the followers. It seems as though she has crossed the threshold of what is accepted within the expressive order of the stable culture. However, the correction process is fast. In correcting the followers, Bella attempts to restore her authority by saying that this [i.e., buying back a previously owned horse based on personal sentiment] is not how it works—implicitly referencing a professional equestrian context. This can also be seen as an example of a restoration of her authenticity. It seems as though her passion is questioned—to speak with Audrezet et al. (2020), she is perhaps even seen as someone with a disembodied authenticity.

The influencer Rebecca has the highest number of posts on Instagram in this study and receives numerous questions about the horses' well-being and how to perform better as a rider. On her Instagram account, Rebecca publishes several similar posts every day describing her horse life. A typical post from Rebecca includes a horse outside a stable, equipped with a saddle and bridle and waiting to be ridden. The rider (most likely Rebecca) has stepped back from the horse to take a picture. The caption presents the horse's name and the kind of work that is planned for the ride.

“XX (the horse's name) first out this morning. Just flatwork today” (20190601)

Rebecca also posts videos, most of which show her working with the horses—very often jumping inside a riding hall. Comments from followers include questions such as: what to do when the horse has back pain, why she does specific exercises and what their function is, how tightly to adjust the reins under the horse's chin, how often she jumps high obstacles, how she trains balance with the horse, and how best to strengthen the horse's canter. The questions center on how the followers can improve as riders and how best to take care of one's horse. Rebecca also has a blog hosted by one of the major equestrian magazines, where she shares knowledge of how to ride and train horses. Rebecca does not seem to provoke her followers. Her social media behavior follows the rules set up for stable culture in cyberspace. Her facework is not questioned and she is perceived as an (passionate) authentic equestrian.

Just like Bella and Rebecca, Fredrik highlights his equestrian skills; he advices his followers on how to exercise their horses to achieve optimal results, what fodder to use, and how to recover from injury as a rider and a horse. It is clear that he is active within an equestrian sports context. The below fieldnotes describe a typical post by Fredrik:

Picture: Fredrik is riding a gray horse with a blue blanket and he is making a winning gesture with a tight fist above his head. Fredrik and the horse wear competition gear.
18,241 likes
Caption: BOOM! Grand slam with my boys today again and XX (horse's name) getting an amazing 9.7 from the test rider putting him first with a collective 91%! All of my five horses have done so well this weekend and I feel so blessed to have such lovely creatures and people around me.
174 comments on how much the followers support him and the way he is riding his horses (20181014).

Fredrik seems keen to emphasize the merits of his horses. He stresses that his horses allow him to perform well. He also gives credit to people working with him. Fredrik's followers praise his facework, and they underline their appreciation of his way of describing the horses' efforts. Just like Bella, he represents a professional stable culture in which horses are bought and sold and must perform well in competitions, however, the rules of the expressive order are not threatened in his posts. The followers are compliant with his treatment of his horses and he encompasses a passionate authenticity. In other typical posts, Fredrik is seen riding and he comments on how he works with his horses. He combines more instructive posts with frequent of pictures of himself, his dog, and Shetland ponies in various fun environments.

Picture: Fredrik is sitting on a stone and is holding two Shetland ponies wearing blankets.
9147 likes
Caption: It's all fun and games until…you get bitten by your own pony
36 comments on how difficult ponies can be, but also how cute these ponies are.
(20181026)

Elin's facework is different from the other influencers discussed. She focuses on liberty dressage, her and the horses' closeness to nature, and a non-competitive context. In a typical post, Elin is seen riding her horse in nature without a saddle or bridle. She wears clothes that seem to belong to another century—long wool skirts and jumpers.

“Join XX (the horse's name) and me riding in the lush green and yellow fields. Mother nature is happy after we got a lot of rain! The fields surrounding the stable are so beautiful right now so we had to sneak up to the flowers to share a glimpse with you guys. And we were careful to not step on the precious plants.”
(20190602).

Elin invites her followers to join her in a nature-romantic relationship with their horses. Her posts feature descriptions of beautiful landscapes in which she rides as well as messages of (environmental) sustainability. Elin's followers admire her and support her way of treating the horses and she does not break the rules of the expressive order of this context. However, Elin's social media communication evinces another stable culture that could have prompted critique from followers who are compliant with Bella, Rebecca, and Fredrik's communication. Likewise, followers of Elin could, theoretically, be opposed to the other influencers' treatment of their horses. It is difficult to know whether the lack of resistance can be explained by the fact that the influencers attract different groups of followers or whether the followers support different stable cultures in different settings.

The performance of horse-knowledge is an important part of the line and face-work of the influencers we have studied. The line and face-work are mostly expressed through horse-related images, captions, and videos. By sharing pictures and comments on their lives, equestrian influencers produce ideas and perceptions of how horses should be cared for. Through face-work, they emphasize themselves as experts in the equine sector—as “real” and passionate horse people—and they stress their own ideas when answering the followers' questions on how to treat and ride horses. The influencers' continuous line is constructed and shaped together with the followers; the understanding and interpretation of the line is constantly co-created through mutual interaction. The line shows that their equestrian lives are a fulltime commitment. This may seem as a paradox in relation to their engagement with social media. They are clearly part of the digital labor market, the line is, however, closely connected to a traditional equestrian sphere.

There seem to be two disparate expressive orders present in relation to horse knowledge. Bella, Fredrik, and Rebecca belong to a traditional equestrian elite, and their online presences showcase a competitive and professional horse life. Horses are seen as tools that can be bought and sold, and the influencers ride other people's horses in competitions. Bella and Rebecca compete in show-jumping, whereas Fredrik competes in dressage (and some jumping). The expressive order is sometimes questioned, as evinced by the critique Bella received for selling a horse. However, Bella discussed this critique and restored the order and her authenticity by telling her followers that this is normal in the professional equestrian world. Equestrian sport is generally seen as expensive, and elite riders are dependent on good—and potentially costly—horses. Even so, riders struggle to earn money as prize sums and sponsorship deals are modest and few. In a study of the conceptions and constructions of elite athletes' economical terms, Hellborg (2019) demonstrates that many elite riders combine their competitive sport performances with selling horses in order to generate an income. Bella's way of restoring the order therefore positions her as an elite rider. Elin's followers seemingly adhere to a distinct expressive order in which the ideas are different and indicate a perception of horses as lifelong companions and friends. This expressive order is not disputed in the posts we have studied. It is also worth indicating that, in contrast to what Wellman (2020) concluded in her study of the bodybuilding culture, the equestrian influencers are not discredited—at least by their followers. On the contrary, they are accepted as part of the equestrian culture. However, it is important to remember that the followers of influencers in our study have chosen to take part in an online culture and are possibly more acquainted with and accepting of its logics than the bodybuilders and trainers that discredit the influencers in Wellman's study. Whether similar criticism would be found among other equestrians is difficult to say, though a study by Broms et al. (2021) demonstrates that the online culture is widely accepted in equestrian sports.

### Advertisement and Sponsorship

Aside from ideas and perceptions of horses and horsey lives, the influencers communicate purchasing decisions. Abidin, referred to in the introduction, underlines that influencers generate income by selling advertising space on their blogs and social media platforms, highly personalized advertorials, and selling wares, with some starting their own brands (Abidin, 2018). The influencers analyzed in this study mix the first and second strategies described by Abidin. None of them sell products or have established their own brands.

Elin seems to have sold advertising space on her blog; she presents several sponsors and uses highly personalized advertisement. She promotes clothes that seems to belong to another century—long dresses and specific jackets in wool—and gear from different companies, and calls herself an ambassador for these companies:

“PS: If you are looking for a belt bag or viking dress, www.xxxxx.no have it in stock! I'm their ambassador” (20190627).

In addition, Elin writes that she wants to collaborate with photographers who can take high-quality pictures of her and her horses. In the pictures of her and the horses, she often wears the clothes she promotes on her blog, giving the advertising a personalized flavor.

The personalization of advertorials is evinced by the influencers' frequent recommendations of products that, supposedly, improve horses' quality of life combined with content that helps to improve riders' equestrian skills. One example is a post by Fredrik promoting a specific veterinary company:

“No one has missed by now that I have a collaboration with xxxx (a digital platform for veterinary advice) and right now they have solved a new function that allows you to get help with printing deworming agents if you have done a draft test, it is very smart now before summer work and things like that start. So, go here on the link, put your animal on *xxxx* and you will get more info” (20190912).

This is an example of a personalized message from Fredrik, the horse expert, who stresses the need for taking good care of one's horse, and that the best way to do so is with the aid of the company he is advertising.

Bella's Instagram account also features personalized advertisements for products for humans and horses. For example, Bella in one post describes using a specific kind of horse feed and tells the reader that this helps her daily work. She instructs her followers to enter a contest to win this horse feed—simultaneously advertising it:

“I'm so grateful for my collaboration with @xxx. It simplifies my everyday life in the stable a lot. Now I give you the chance to win” (20190601).

Advertisements featuring contests or special discounts are common. For example, one post on Rebecca's Instagram shows a picture of a gift voucher (14 June 2019). In the caption, she informs her followers that one of them has won this voucher (3000 SEK) for a specific equestrian store. Another typical post from Rebecca is:

“Want to promote this fantastic product from @xxxx You can take the opportunity to buy it now (and lots of other things) now at @xxxx when they have 10% all weekend” (20190628).

Clearly, Rebecca wants to communicate that she herself uses the product—while simultaneously trying to promote it to others, thereby influencing their purchasing decisions.

In the examples above, the influencer presents a commercial product or agent—in other posts the commercial interest is less clear. Social media advertising exists in a legal grey area and has prompted new national advertising regulations. According to Swedish Advertising Ombudsman (Reklamombudsmannen, RO) practice on social media—not least Instagram—high demands are placed on the consumer to be able to identify advertising as such (Reklamombudsmannen, 2019). In one post, Bella informs her followers about the wonders of a specific horse rug; in another, Fredrik tells his followers about saddle pads and which one he prefers. In addition, followers ask the influencers about good products and which ones they prefer. In another picture, Fredrik is seen in front of a poster advertising an insurance company. Neither the picture nor the accompanying caption indicates whether this is advertising. Instead, followers may feel as though the picture was uploaded by one of their friends, who just happened to be standing in front of a poster. However, the same insurance company's logo is printed on Fredrik's vest, which may suggest that he is sponsored by the company. Their marketing strategy can be described as containing an absolute authenticity (Audrezet et al., 2020).

The lines of advertising are also blurred when Bella presents clothes on her media platforms. The advertising collaborations are sometimes clearly indicated, with the posts marked #collaboration. However, Bella also posts pictures and videos (both in the same post) of herself with beauty products and wearing an item of clothing, using an @ to tag the designer. Some of Bella's followers ask: “Bella; are you doing commercials for these brands?” Bella does not answer these direct questions. In these posts, Bella represents a more disembodied strategy, disconnecting her from the horse life.

The influencers we have studied seemingly apply two of Abidin's (2015) ways of generating income in the digital world. It is difficult to know whether their income from this labor is substantial. Bella, Rebecca, and Fredrik also appear to combine traditional ways of making money in the equine sector by selling horses and riding other people's horses. Once again, their face-work is built on their belonging to the equine sector. Of these three, Bella stands out as an influencer working in several different sectors; the equine sector as well as the beauty sector (promoting cosmetics and clothes). It is less clear how Elin generates income.

Intimacy and a common love of horses creates trust between the influencers and their followers. As suggested by Stubb and Colliander (2019), the perceived closeness and common understanding generated through an acceptance of face-work, line, and the expressive order build a solid base for affecting others' purchasing decisions. The forms of personalized advertisement and the blurred lines between seemingly friendly recommendations and what is bought and sold makes it difficult to grasp the influencers' commercial power. Hence, the expressive order seems to be quite stable.

Abidin (2018) underlines that influencers have impacted the shift from an attention economy to an affection economy. She indicates that it is lucrative for influencers to cultivate their self as a brand and market products through the lens of their persona, rather than through a company. As demonstrated above, the influencers in this study use their face-work to generate income. However, the “raw aesthetic of an amateur” and calibrated amateurism, which Abidin observes is recurrent among influencers, is not reflected by the influencers we have studied (Abidin, 2018, p. 91–97). Many pictures and videos appear to have been captured by professional photographers; Elin even invites professional photographers to work with her, and in interviews Fredrik stresses the importance of posting beautiful pictures. Rebecca and Bella have another face-work. In some of Bella's posts, it is clear that she takes the pictures herself—she takes pictures *of* herself and advertises using selfies. Rebecca photographs all the horses she rides. The pictures are plain in comparison to those posted by Fredrik and Elin—constructing her authenticity.

Furthermore, it is clear that the products *per se* are not what is most important. Instead, the influencer's choice of a particular product is decisive. If the influencer were to change brand and product and choose, for example, another saddle or type of feed, followers would likely mirror the influencer's choice of brand. A follower is faithful to the influencer's choice rather than the brand itself, making influencer advertisements and sponsorship part of the affection economy (Abidin, 2018).

## Concluding Discussion

People's understanding and commitment to their hobbies and sports develop in relation to others and shared perceptions are crucial for the relationship between influencers and their followers. Influencers wield considerable power over the economy, impact, and development of the contemporary sports landscape. This article has aimed to explore how social media influencers within equestrian sports perform and communicate with their followers in order to uncover how perceptions, ideas, and perceived knowledge are formed and transferred in this sport. Goffman's concepts face, face-work, line, and expressive order have been used to study and analyze the influencers' performance and communication on social media. Our findings indicate that the equestrian influencers build trust and intimacy and construct authenticity in relation to their sport. Their face-work and line produce images of them as experts in the sector.

The influencers' posts do not stand on their own as followers add comments, share experiences, and give the influencers advice, and vice versa. In the accounts we have followed, influencers and followers present few challenges and disagreements about their life choices, and rarely correct each other. In the cases where the expressive order appears to be questioned, the influencers have the power to correct their followers as exemplified by Bella's way of handling the sale of her horse. The followers can resist by (threatening to) leave the influencer. When the followers accept these corrections and show gratitude, they can continue within the common expressive order. The influencers mainly focus their communication on horse-related issues, with the exception of Bella, who discusses life choices in general and advertises clothes and cosmetics. There are, however, some differences in the influencers' presentation of human-horse relationship. Bella, Fredrik, and Rebecca all highlight a competitive equestrian context, hard work, and improved skills while Elin emphasizes the human-horse relationship, riding in a natural environment, and sustainability. All of the influencers we have studied mostly receive positive feedback from their followers for their way of handling their horses' lives. It seems as they are accepted as equestrians as well as influencers—in difference from what Wellman demonstrated in her study of the bodybuilding culture (2020). It is possible that the difference can be explained by the fact that the online culture is more widely acknowledged and accepted within equestrian sport (cf. Broms et al., 2021). The expressive order is seldom questioned—and therefore restoration is rarely needed.

Common understandings create intimacy between the influencers and their followers. The influencers can utilize this closeness to affect followers' purchasing decisions, and it is possible that this impact is even stronger in a context where the expressive order is seldom challenged and where the influencers' marketing strategies (mostly) encompass an absolute authenticity (cf. Audrezet et al., 2020), than in a context where decisions are constantly questioned. The intimacy between influencers and followers must be problematized as it is likely to make the commercial endorsements less visible and more convincing.

However, the extent to which this intimacy is shared is questionable. Influencers choose which comments to respond to, answering comments that strengthen their face-work. As shown above, this is exemplified by Bella's refusal to answer questions about whether or not she is paid to advertise various brands. This is an exercise of micro-power as the influencer decides the rules of the game, thus consolidating the order of power between the influencer and the followers. Magnusson (2017) states that Instagram is not dependent on social interaction but can be used as a mass medium; as a megaphone to proclaim a message, and that the perceived closeness and social community can thus be as apparent as in parasocial interaction. However, there is some ambivalence here; a significant difference is that the distance between influencers and followers has been reduced by today's possibilities for two-way communication between them. Thereby, even if the followers may never meet the influencer in real life, mutual connection and cultural intimacy are much stronger in the social media landscape than in the parasocial relations described by Horton and Wohl (1956).

Researching social media entails challenges, not least in terms of selection process and choice of focus areas since the social media landscape is difficult to overview. One limitation of this study is that emojis and other characters that complement traditional language and pictures were not included. As the semiotics of social media are constantly changing it would be fruitful to scrutinize a greater variety of signs, pictures, and languages in future studies. This may enable a deeper understanding of the interaction order and face-work on social media, not least since emojis are frequently used in communication between influencers and followers (Abdalla and Baskerville, 2019). Another limitation is that we have not problematized Goffman's lack of temporality, as discussed by Kerrigan and Hart (2016). They state that digital users have both temporal and contemporaneous multiple selves. Our study focuses on the contemporaneous selves, though it would be fruitful for future studies to include both perspectives.

A third limitation is that we chose to follow influencers based in Sweden and Norway. It is difficult to know whether the results would have been different if we had compared an international sample of equestrian influencers. Moreover, interviewing the four investigated influencers may have generated a deepened understanding of their performances on social media. Despite the limitations presented above, our study explains the interaction between influencers and devoted followers and enables a better understanding of how social media works. Increased knowledge of the influencers' impact on the landscape of sports is essential in relation to influencer-follower dynamics, perceived authenticity, generation of trust, and the impact on consumer behaviour.

## Data Availability Statement

The original contributions presented in the study are included in the article/supplementary material, further inquiries can be directed to the corresponding author/s.

## Author Contributions

AR, SH, and LB contributed to the research idea, the design of the study, and contributed to the media analysis of the study. AR wrote the first draft of the manuscript. All authors contributed to manuscript revision, read, and approved the submitted version.

## Conflict of Interest

The authors declare that the research was conducted in the absence of any commercial or financial relationships that could be construed as a potential conflict of interest.

## Footnotes

^1^https://www.instagram.com/dagens_nyheter/?hl=sv (Accessed December 10, 2020).

^2^https://www.instagram.com/aftonbladet/?hl=sv (Accessed December 10, 2020).

^3^https://www.instagram.com/iamzlatanibrahimovic/?hl=sv (Accessed December 10, 2020).
